# Radon Biomonitoring and microRNA in Lung Cancer

**DOI:** 10.3390/ijms21062154

**Published:** 2020-03-20

**Authors:** Rakhmet Bersimbaev, Alessandra Pulliero, Olga Bulgakova, Kussainova Asia, Akmara Aripova, Alberto Izzotti

**Affiliations:** 1 Department of General Biology and Genomics, Institute of Cell Biology and Biotechnology, L.N.Gumilyov Eurasian National University, Nur-Sultan, Akmola 010008, Kazakhstan; ribers@mail.ru (R.B.); ya.summer13@yandex.kz (O.B.); assya.kussainova@gmail.com (K.A.); aripova001@gamil.com (A.A.); 2 Department of Experimental Medicine, University of Genoa, I-16132 Genoa, Italy; izzotti@unige.it; 3 IRCCS Policlinico San Martino, 16132 Genoa, Italy

**Keywords:** radon, lung cancer, microRNA, biomonitoring

## Abstract

Radon is the number one cause of lung cancer in non-smokers. microRNA expression in human bronchial epithelium cells is altered by radon, with particular reference to upregulation of miR-16, miR-15, miR-23, miR-19, miR-125, and downregulation of let-7, miR-194, miR-373, miR-124, miR-146, miR-369, and miR-652. These alterations alter cell cycle, oxidative stress, inflammation, oncogene suppression, and malignant transformation. Also DNA methylation is altered as a consequence of miR-29 modification induced by radon. Indeed miR-29 targets DNA methyltransferases causing inhibition of CpG sites methylation. Massive microRNA dysregulation occurs in the lung due to radon expose and is functionally related with the resulting lung damage. However, in humans this massive lung microRNA alterations only barely reflect onto blood microRNAs. Indeed, blood miR-19 was not found altered in radon-exposed subjects. Thus, microRNAs are massively dysregulated in experimental models of radon lung carcinogenesis. In humans these events are initially adaptive being aimed at inhibiting neoplastic transformation. Only in case of long-term exposure to radon, microRNA alterations lead towards cancer development. Accordingly, it is difficult in human to establish a microRNA signature reflecting radon exposure. Additional studies are required to understand the role of microRNAs in pathogenesis of radon-induced lung cancer.

## 1. Introduction

Lung cancer is the most common killing cancer in humans [1]. The World Health Organization (WHO) has listed lung cancer as the leading cause of death worldwide. It was estimated that at present there are 8.2 million deaths annually [2]. By 2030, it is estimated that the number of lung cancer deaths will rise to 10 million per year [3]. Like the other types of cancer, lung cancer is a multifactorial disease. Genetic and epigenetic changes in the cell and environmental factors play an important role in its pathogenesis. Cigarette smoking is the main risk factor but other factors are responsible for the increasing burden of lung cancer in non-smokers mainly including radon and its daughter decay products, airborne pollution, and passive smoke [4]. Radon is the number one cause of lung cancer among non-smokers, according to the Environmental Protection Agency (EPA) estimates. Overall, radon is the second leading cause of lung cancer being responsible for approximately 21,000 lung cancer deaths every year in USA [5]. The WHO considers radon as the second leading cause of lung cancer after tobacco smoke [4]. Other exogenous factors include exposure to arsenic and asbestos [6], xenobiotic pollution, heavy metals, alcohol, and malnutrition [7].

The epigenetic basis of lung cancer is related primarily to changes in the profile of microRNA (miRNA). MiRNAs are a class of small single-stranded non-protein-coding RNAs that play important roles in different cellular processes including cell development and proliferation, differentiation, growth control, and apoptosis [8,9,10]. MiRNAs are generally composed of 18–25 nucleotides in length that are highly conserved in evolution and highly specific in tissues [11]. MiRNAs are involved in the regulation of target genes at the post-transcriptional level, miRNAs can covalently bind to complementary sequences of the 3′UTR region of the mRNA and thus inhibit translation. Recently, a large amount of evidence has been accumulated on the involvement of miRNAs in the carcinogenesis of various malignant tumors, including lung cancer [12].

Recent data indicated that miRNAs are engaged in the regulation of cellular processes induced by radiation and, consequently miRNAs can potentially be used as biomarkers to assess the degree of exposure to radiation in humans [13]. MicroRNAs are massively dysregulated during lung carcinogenesis induced by cigarette smoke [14] and by other environmental airborne lung carcinogens [15]. In humans, the expression profile of a number of miRNAs, in bronchial epithelium BEAS2B cells, have been altered upon exposure to radon [16]. Accordingly, a major problem limiting the secondary prevention of lung cancer and its early diagnosis is the lack of predictive intermediate biomarkers that may preliminarily identify high-risk subjects.

In this review, we consider the change of miRNA profiles as related to radon exposure focusing on the current knowledge of epigenetic changes associated with radon exposure and lung cancer.

miRNA may be proposed as early diagnostic tool in cancer to identify high-risk subjects and early cancer stages by liquid biopsy [17].

Accordingly, the definition of a radon related miRNA signature represents a milestone for the secondary prevention of radon associated lung cancer in exposed populations.

## 2. Decay of Uranium: Radon as a Radiation Factor of the Environment

**Figure 1:**
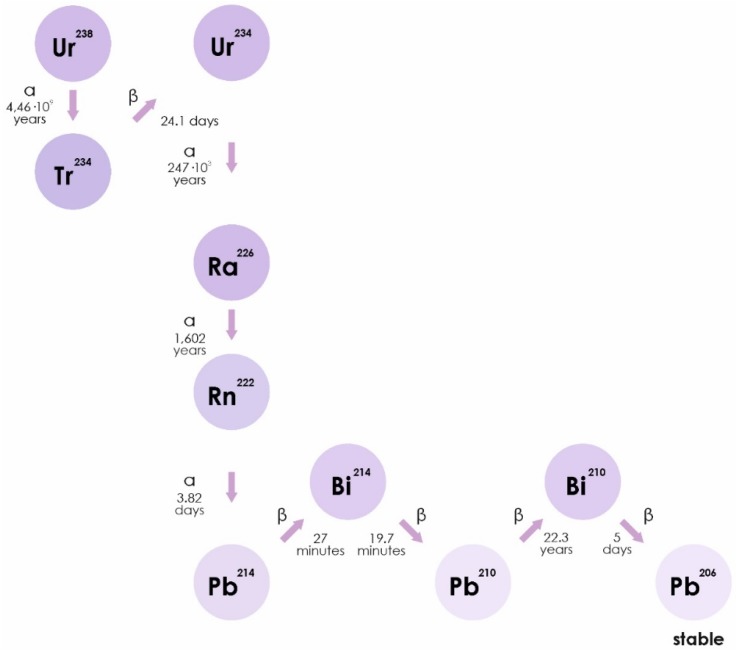
The isotope ^222^Rn is a direct decay product of radium-226 (^226^Ra), which is part of a decay series beginning with uranium-238 (^238^U). Thorium-230 and -234 (^230^Th and ^234^Th) are also part of this decay series. ^222^Rn includes a series of decay: ^222^Rn (α)→^218^Po (α)→^214^Pb→^214^Bi→^214^Po (α)→^210^Pb, and is any combination of half-lived radon progeny.

Radon is a chemically inert radioactive gas, occurring naturally as an indirect decay product of uranium. Radon isotopes, ^222^Rn, ^220^Rn, and ^219^Rn, are the central members of each natural radioactive chain [18]. The WHO estimates that radon accounts for half of the average annual natural radioactive background of the earth. Radon can be found in soil, water, and air in different concentrations (https://www.who.int/ru/news-room/fact-sheets/detail/radon-and-health). Radon isotopes are unstable. They disintegrate to form isotopes of polonium (^218^Po), lead (^214^Pb), and bismuth. The half-life of radon can range from a minute (^220^Rn) to several days (^222^Rn) and is accompanied by the release of alpha particles (Figure 1) [19].

Radon (Rn) is an established natural radioactive carcinogen emitted from rock, soil, and concrete and an indoor air quality concern, especially in buildings with low outdoor air exchange rates. A recent estimate put the radon contribution at 14% of total lung cancer deaths. The main source of radon in the air and living quarters is its passive diffusion from the soil. Radon migrates out of soil and rock into the surrounding air, resulting in accumulation in poorly ventilated or closed areas. Such areas represent the primary environments in which humans are exposed to radioactivity from radon [20]. Radon permeates through cracks in the Earth’s crust and accumulates in the lower atmosphere. Increase concentration of radon in the air is observed in regions rich in uranium deposits, as well as near to uranium mines [21,22].

More than two-thirds of the world’s uranium production is in Kazakhstan, Canada, and Australia (https://www.world-nuclear.org/information-library/nuclear-fuel-cycle/mining-of-uranium/world-uranium-mining-production.aspx). Kazakhstan produces the largest share of uranium from mines (41%), followed by Canada (13%), Australia (12%), USA, France, Germany, and Spain. In these countries, there is a high concentration of radon in the atmosphere and water sources [23].

Radon pollution is highly concentrated in selected areas. However, the presence of natural uranium occurs almost everywhere, although to various extents. Accordingly, all humans are exposed to radon to some degree. Radon penetrates into indoor environments from foundation, especially when indirect contact with earth, or from building materials. Radon dilution depends on the number of air renewal cycles, radon concentration being increased in houses highly isolated because of thermic reasons. Also tap water and cooking gas represents possible sources of radon pollution. The maximum threshold of radon in most countries is 200 Bq/m^3^, however, some studies report an elevated lung cancer risk at radon levels as low as 100 Bq/m^3^ [23,24], while others consider exposed subjects at >50 Bq/m^3^.

## 3. Toxic Effects of Exposure to Radon in Human

The first comprehensive study of the toxic effects of radon exposure on human health were reported in a book “Health Risks of Radon and Other Internally Deposited Alpha-Emitters: BEIR IV” [24]. This report described the relationship of radon with the development of lung cancer in uranium miners. According to this BEIR IV report, the excess of relative risk per 100 WLM (Working Level Month) was 1.3 WLM, defined as a cumulative exposure of an individual at a concentration of 1 WL for a working month of 170 h. Working Level (WL) is defined as any combination of short-lived radon progeny in 1 L of air that results in the ultimate release of 1.3 × 10^5^ MeV of potential energy from alpha particles. One WLM is approximately equivalent to the dose received by a person who lives for a year in a dwelling with a radon concentration of 227 Bq/m^3^ [25]. Five years after the BEIR IV report, in the 1993 the ICRP (International Commission on Radiological Protection) published a further report [26], where the excessive relative risk per 100 WLM was similar to the result of BEIR IV and equal to 1.34 WLM. Then, in 1999, it was published in the BEIR VI report and, in 2006, in the UNSCEAR (United Nations Scientific Committee on the Effects of Atomic Radiation) report. According to the UNSCEAR report, the excess relative risk per 100 WLM was 0.59. It was demonstrated that miners exposed at a younger age and exposed to relatively low radon concentrations had a higher percentage increase in lung cancer death rate per WLM, compared to other miners. Taking into account these results of Reports by different Commissions, the recommended maximum reference level of indoor radon was lowered from 600 Bq/m^3^, which corresponds to an annual effective dose of 4 mSv at workplace and 14 mSv at home [25].

The role of radon and its radioactive decay products as human carcinogens has been established by epidemiologic studies in humans [27]. It is well demonstrated that radon induces DNA mutations and subsequential functional damage [28]. The high-linear energy transfer of α-particles emitted by radon and radon decay products can directly attack genomic DNA and cause mainly double-strand breaks in DNA [29].

In comparison with the damaging effects of β-, and γ- radiation, alpha particles cause around 40 times more severe radiation damage. Decay of α-particles results in the ejection of electrons from water, generating several oxidative reactive species leading to cellular damage by hydroxyl radical attack [30]. In addition, overproduction of reactive oxygen species (ROS) in the lungs caused by persistent radon exposure may cause oxidative stress, leading to pulmonary inflammation, tissue damage and eventually to chronic lung diseases such as chronic obstructive pulmonary disease (COPD), pulmonary fibrosis, and lung cancer [31]. Exposure to rocks emitting radon gas at a level of four times higher than average global outdoor levels can significantly affect gene expression levels in cultured human lung cells [32].

## 4. Radon Exposure and Lung Cancer: Epidemiology and Biomonitoring.

The relationship between radon and lung cancer is well documented by epidemiologic studies documenting that 70% of lung cancer deaths among uranium miners is attributable to radon exposure [6,33,34]. Uranium miners have a remarkably high genomic damage in blood lymphocytes [34] as well as a high risk of developing lung cancer [35]. Epidemiological monitoring of 68,000 uranium miners from including Germany, USA, Canada, Czechoslovakia, and countries highlighted that 2700 died because of lung cancer. Radon related lung carcinogenicity does not only affect occupational exposure but also the general population. Indeed, it has been estimated that 15,000 deaths from lung cancer occur due to radon exposure in USA and more than 2500 in UK (https://www.who.int/news-room/fact-sheets/detail/radon-and-health). Also in Czech Republic, undergoing high levels of natural pollution, radon was reported as the second most important risk factor for lung cancer, after cigarette smoking [36].

The main of source of radon income from indoor air (>95%), only a minor fraction from other mainly sources including water (1%). Of this amount, radon is mainly released from contaminated water because of running water activities, including cleaning, bathing, showering, hot-tube water bath. Only 0.1% of this amount penetrate in the body because of drinking, the stomach being the most exposed organ (https://www.who.int/news-room/fact-sheets/detail/radon-and-health).

Occupationally radon-exposed workers from Carlsbad region of the Czech Republic undergo increased levels of total DNA damage with increased levels of chromosomal breaks. Conversely, in spa personnel exposed to radon, the length of exposure was associated with a non-significant decrease of DNA damage. This supports the idea of the gradual adaptation of the organism to new conditions [36].

**Figure 2:**
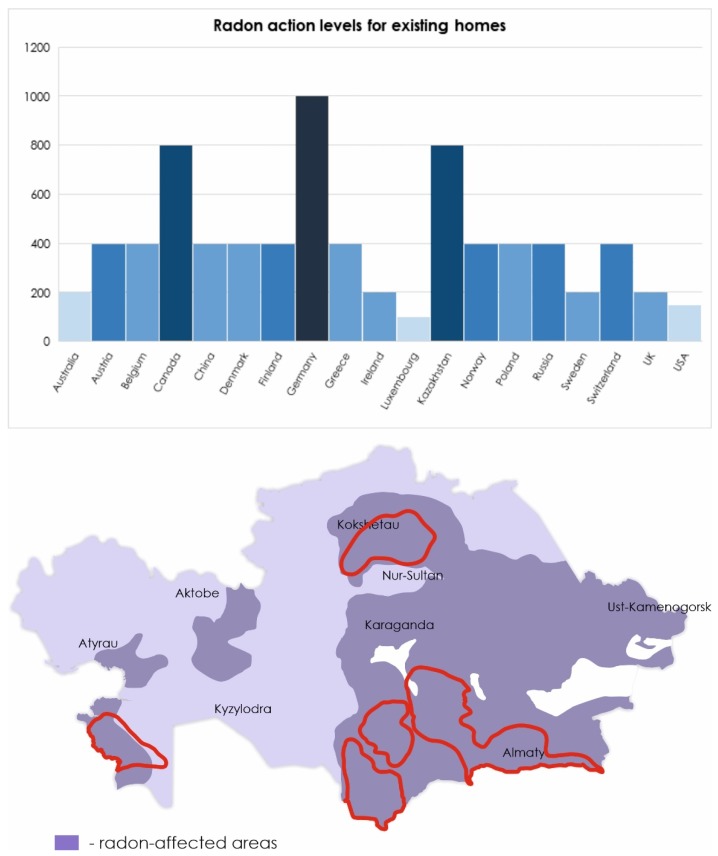
Radon levels in different countries according to the World Health Organization (WHO) handbook [5]. Level of radon in Kazakhstan presented according to [22,37]. The borders of natural uranium mining zones are highlighted in red.

As world statistics show, in countries where the mining industry is developed, lung cancer occupies a leading position among other cancers. According to the International Agency for research on cancer in 2018 in Kazakhstan, 12.6% of all cases of cancer are lung tumors, in Canada (10.2%), in Australia (6.7%), the United States (10.7%), Germany (11%), and Spain (10.1%) (http://gco.iarc.fr). The dimension of the environmental Radon pollution in Kazakhstan is outlined in Figure 2.

The WHO has set a standard annual average radon concentration of 100 Bq/m^3^ (https://www.who.int/news-room/fact-sheets/detail/radon-and-health). However, uranium-exporting countries increased this value by defining a normal level of radon in residential premises equal to 200 Bq/m^3^ (https://online.zakon.kz/document/?doc_id=1017332#pos=1;-119), (https://www.canada.ca/en/health-canada/services/environmental-workplace-health/radiation/radon/government-canada-radon-guideline.html). In the Eastern part of Kazakhstan, there is an increase in the incidence of lung cancer by 3.4% per year. As mentioned above, high radon activity was detected in the East Kazakhstan region. It can be assumed that the increase in the incidence of lung cancer is due to exposure to radon [1,37].

A meta-analysis of combined studies that showed a direct link between household radon exposure and lung cancer found that 6.9% of lung cancer cases in Canada were associated with household radon exposure. The relative risk of lung cancer with prolonged exposure to radon in the population is 11.3% [38].

The results of the study conducted among uranium miners of Wismut Company (East Germany) indicate an increased risk of lung cancer under long-term exposure to low doses of radon, which suggests a high risk of lung cancer among the general population [35]. Indeed, a study showed that exposure to low doses of radon, namely more than 37 Bq/m^3^, is a risk factor for lung cancer for the Spanish population as a whole [27].

Thus, many studies conducted independently at different time periods identified household radon as a serious risk factor for lung cancer.

It was estimated that approximately 17% of cases of lung cancer in Alberta are associated with exposure to radon (70 Bq/m^3^) [39]. Hassfjell et al. studying the effects of radon in living quarters on lung cancer risk, reported that 12% of all lung cancer cases per year are radon-dependent at an average indoor concentration of 88 Bq/m^3^ [40]. A small risk of developing a lung tumor with prolonged exposure to radon (100 Bq/m^3^) is shown in a study carried out by Baysson et al. [41].

Data from Lorenzo-González et al. showed that natural radon is an important risk factor for lung cancer in non-smoking patients exposed to radon levels above 200 Bq/m^3^ [42].

It was observed that a tendency to increase the risk of lung cancer from the concentration of radon in living quarters. This is confirmed by the results of the Torres-Durán et al. study, which showed the dependence of the degree of risk of radon-induced lung cancer on the dose of radon exposure. They have determined that individuals exposed to radon exposure more than 200 Bq/m^3^ had a higher risk of lung cancer than those exposed to low doses (<100 Bq/m^3^) [43]. In addition, Zhang et al. showed a direct correlation between the dose-dependent effect of radon exposure and lung cancer growth. It has been proven experimentally that an increase in the concentration of radon in living quarters for every 100 Bq/m^3^ leads to an increase the risk of lung cancer to 7% annually [44]. Kreuzer et al. demonstrated a clearly increased excess risk of lung cancer at low cumulative radon exposures [45]. Moreover, it was shown that excess relative risk (ERR) for lung cancer mortality per unit of cumulative radon exposure in WLM was 0.006 (95% confidence interval (CI): 0.003; 0.010) based on 1254 lung cancer deaths [45]. According to Yarmoshenko et al. the relative risk (RR) of radon-induced lung cancer mortality was 0.026 (90% CI: (0.11–0.17)) and 0.83 (90% CI: (0.52–1.12)) per radon concentration 100 Bq/m^3^ for males and females, respectively [46]. Another study showed that lung cancer mortality was directly associated with locations with high levels of radon. The mean values of the radon concentrations in homes of deceased’s participants were 217.1 and 247.8 Bq/m^3^ [47]. An increase of radon level was associated with a 2.62% increase (95% CI 2.52%; 2.73%) in total mortality [48].

## 5. Radon Exposure and Lung Cancer: Pathogenic Mechanisms (Genotoxicity, Inflammation, and Oxidative Stress)

Radon, as alpha emitters, directly cause genotoxic and clastogenic damage at the site of absorption. However, adverse effects on distant organs are not conceivable this radon gas genotoxicity being primarily addressed to bronchial epithelia and lung parenchyma. Effects on distant organs could be only related to the absorption on nanoparticles able to cross the capillary endothelium in the lung. However, insofar this situation has been never demonstrated. Further to direct genotoxic damage, radon displays other indirect pathogenic effects.

Lung tissue was severely injured when exposed to radon as demonstrated by pathological diagnosis and immunohistochemical analysis [49]. Accordingly, the pathogenic cascade event triggered by radon is genotoxic damage, apoptosis, and inflammation.

Radon isotopes enter the human organism through the lungs, where further decay occurs emitting ionizing radiation causing oxidative damage to DNA, proteins, and lipids [50]. Accumulation of such damages in a cell, contribute to push it to malignant transformation [51].

Emerging numbers of cohort studies indicate the carcinogenic potential of radon. Radon enters the body through the respiratory system. Therefore, the main target of its toxic effects are lung cells. Radon-induced damage leads to malignant degeneration. Likewise, cumulative radon exposure may contribute to increased tumor mutation burden in never-smoker patients with lung cancer, and the mutational signature was associated with defective DNA mismatch repair. Examining the mutational landscape in 439 non-smoker lung cancers a higher frequency in high (i.e., >45 Bq/m^3^) vs. low exposed Radon patients was found for mutations targeting the DNA damage response/repair machinery (*ATR*, *ATRX*, *BARD1*, *RAD50*, and *SMARCA4*), histonedeacetylase2 (*HDAC2*), and inhibitor of nuclear factor kappaB kinase subunit epsilon (IKBKE), as well as *EGFR-* and *TP53* [52]. It was shown that radon-induced lung cancer susceptibility is related to genes involving to DNA dealkylation [53]. Radon exposure was found to be associated with exon 19 *EGFR* and *ALK* mutations in 393 never-smokers lung cancer cases [54] as well as increased TP53 mutations comparing 83 non-smokers vs. 250 smoking lung cancers [55].

Genetic factors are also significant contributors to the pathogenesis of lung cancer. First-degree relatives of patients with lung cancer are at increased risk, even after adjusting for smoking habits. This pooled analysis included 24 case-control studies in the International Lung Cancer Consortium. Data from 24,380 lung cancer cases and 23,305 healthy controls were analyzed. The association was strongest for those with a family history in a sibling, after adjustment [56]. The carcinogenic effect is caused by the accumulation of genetic polymorphisms [57], chromosomal abnormalities and mutations in tumor suppressor genes [58]. The *ERCC1* rs3212986 GT-TT polymorphism is remarkably associated with an increased risk of developing lung cancer in case of exposure to radon. A similar situation occurs for, homozygous GSTM1 deletion in non-smokers exposed to radon [59]. Polymorphisms of DNA repair genes (*APE*, *XRCC1*, *OGG1*, *ADPRT*, *XpC*, *XpD*, *XpG*, *Lig4*, and *NBS1*) have been also associated with an increased risk of chromosome aberration in radon-exposed children. Cytogenetic damage suggests that these three SNPs (rs13181, rs17655, and rs1136410) may be considered radio-sensitivity markers. Although, several studies have been demonstrated the role of candidate genes for developing lung cancer in non-smokers. However, the genetic determinants for susceptibility in lung cancer in non-smokers with residential radon exposure are still uncertain [60].

## 6. Epigenetic Factors in the Development of Cancer

Epigenetic factors include DNA methylation, modification of histones, and miRNA molecules. In eukaryotes, DNA methylation occurs at CpG sites, which are included in the sequence of many gene promoters. DNA methylation changes are observed in cancer. Hypermethylation of CpG islands located in the promoter regions of tumor suppressor genes leads to the inhibition of transcription and, as a consequence, to the lack of oncosuppressor protein [61].

Histone acetylase overexpression, which catalyzes the removal of acetyl groups on the tail of histones, is another mechanism of transcriptional repression that leads to chromatin remodeling and genome instability in lung cancer [62].

miRNAs play important roles in different cellular processes including cell development and proliferation, differentiation, growth control, and apoptosis [9,11]. It was shown that miRNAs can regulate the expression of at least 30% of genes that control various biological functions [63,64]. miRNAs regulate the expression of its target genes by binding to the 3′-untranslated region (3′-UTR) of the mRNAs, and thus inhibit translation and promote the degradation of target mRNA in various physiological and pathological processes. miRNA maturation process [65,66] finally results in the incorporation of mature miRNA short single strand into the RNA-induced silencing complex (RISC) selectively activating RNAses to destroy targeted messenger RNAs [66]. Each miRNA can regulate multiple target genes, while the specific target mRNA can also regulated by multiple miRNAs at the same time. Therefore, miRNA are involved in multiple biological processes, including gene regulation, cell development and proliferation, maintenance of cell differentiation, growth control, and apoptosis. Now it is well estimated that one-third of human genes are regulated by miRNAs and miRNAs play a key role in regulatory mechanisms of many cell function [10,11,63].

In recent decades, accumulating evidence has suggested that some miRNAs can function as oncogenes or tumor suppressors, so they can regulate several genes that play important roles in tumorigenesis [10,11,64]. Studies have found that many miRNAs have abnormal expression in tumors and play a key role in controlling the occurrence, development, metastasis, and drug resistance of cancers. They have formed a complicated regulatory network, thus playing roles as oncogene or tumor suppressor gene in tumor genesis and development.

MicroRNA also play an important role in regulating various target genes associated with cancer, including lung cancer. Under their control are the mechanisms of proliferation and cell death, the shutdown of oncogenes and oncosuppressors, the cell cycle, and the immune response. Recently, 4719 human miRNA molecules are known (http://www.mirbase.org/) of which more than a thousand are associated with various diseases, including malignant tumors [67].

It is noted that the change of miRNA profile in carcinogenesis is regulated by the following mechanisms:

Often malignant transformation occurs as a result of various chromosomal aberrations. The in silico analysis showed that miRNAs genes are localized directly to the regions of the chromosome where such changes occur [68]. As already mentioned above, ionizing radiation caused by the decay of radon isotopes leads to double-strand breaks of DNA, which as a result can lead to chromosomal rearrangements and as a consequence to a change in the level of certain miRNA molecules. An estimated 10% of miRNA expression is controlled by DNA methylation [69].

Variations miRNA profiles are observed in various bronchopulmonary diseases, such as pulmonary fibrosis, bronchial asthma and COPD [70]. These diseases are known to increase the risk of lung cancer in patients [71]. It is worth noting that the basis of these diseases and lung cancer are genetic predisposition and environmental factors [72].

Carcinogenesis is a complex multistage process of malignant cell degeneration and tumor development. Each stage of transformation is controlled by epigenetic regulation of the processes occurring in the cell. A change in the miRNA profile has been demonstrated in most cancer cases [69].

Overexpression of miR-221-3p in non-small cell lung cancer (NSCLC) has been shown targets p27 and promotes the cell cycle progression of NSCLC cells [73].

Similar data were obtained, regarding miR-19, which inhibited CBX7 expression, which led to activation of proliferation, migration, and invasion of NSCLC cells [74].

The results of the study Feng et al. indicate the oncosuppressive role of miR-34b-3p in NSCLC. This miRNA is able to target CDK4, which leads to inhibition of proliferation and apoptosis of cancer cells [75].

miRNA-374b was markedly downregulated in the blood and tumor tissues from NSCLC patients. Conversely, overexpression of miRNA-374b markedly reduces the viability of NSCLC cells, activating apoptosis and inhibiting tumor formation. Western blot analysis showed that miRNA-374b regulates tumor progression by regulating the p38/ERK signaling pathway [10].

It was known the role of Wnt5a-S protein in the processes of tumor formation and growth. Liu et al. positioned WNT5A as a target for miR-1253 [76]. In vitro and in vivo experiments showed that overexpression of miR-1253 significantly inhibited proliferation, migration, and invasion of NSCLC cells, while inhibition of miR-1253 had the opposite effect.

Furthermore, it was described that the change in miRNA expression in cancer leads not only to tumor progression, but also to metastasis [11]. A change in the miRNA profile is associated with the development of tumor processes in the lung. miRNAs exhibit oncogene properties, maintaining a high level of proliferation and development of the tumor and oncosuppressors, inhibiting the division and invasion of cancer cells [11]. MicroRNAs are massively dysregulated during lung carcinogenesis induced by cigarette smoke [14,15] and by other environmental airborne lung carcinogens [16]. miRNAs dysregulation in lung may be released extracellularly in body fluids, including blood, during the different stages of carcinogenesis such as development of micro-adenomas, adenomas, and malignant tumors [77]. Indeed, miRNAs play a pathogenic role in cancer only when the silenced oncogene is mutated and the extracellular release of miRNAs corresponds to a cancer related event and not to an adaptive response to carcinogen exposure. Carcinogen exposure blocks the miRNAs maturation by interfering with DICER function thereby mainly inducing release of free miRNA precursors [77]. Conversely, miRNAs released from cancer cells are contained in exosomes and micro-vesicles that are used to communicate with other cells. This mechanism blocks specific immunity and activates epithelial–mesenchymal transition [78], induces skeletal muscle cachexia to provide metabolites for cancer development and activates inflammation and tumor associated macrophages to promote cancer growth [78]. Indeed, miRNA overload activates TL3 receptors in lymphocytes triggering cytokine production, protease release, leukocytes recruitment and inflammation [79]. Release of extracellular vesicles plays a pathogenic role in the lung damage induced by cigarette smoke [80].

miRNAs themselves also contribute to inhibition of methylation of CpG sites. For example, miR-29 targets the mRNA DNA methyltransferases DNMT 3A and-3B, resulting in a reduced methylation profile [81].

Transcription factors can induce the transcription of pri-miRNAs. This regulation is often observed in cancer cells. It was shown that p53, c-Myc and EGF are the main transcription factors [82,83] participating in this process. Changes in the miRNA profile in various pathological conditions characterize them as a new generation of epigenetic biomarkers and a potential therapeutic target in the future [84].

## 7. Epigenetic Factors in the Development of Radon-Induced Lung Cancer 

miRNAs are engaged in the regulation of cellular processes induced by radiation and, consequently, miRNAs can potentially be used as biomarkers to assess the degree of exposure to radiation in humans [11,85,86].

Collectively, 1000 dysregulated non-coding RNA (ncRNA) and miRNA transcripts were found due to radon expose and these long non-coding RNA (lncRNA) play an important role in lung damage following radon exposure [87].

miRNAs play an important role in regulating various target genes associated with lung cancer. Researchers demonstrated that chronic radon exposure up-regulated the expressions of miR-34a and enhanced cellular apoptosis in a time-dependent manner. Indeed, chronic exposure to radon produced up-regulation of miR-34a gene which subsequently enhanced apoptosis in BEAS-2B cells [11].

ncRNAs exert biological functions by interacting with RNAs, proteins, and DNA. Lung damage associated with radon exposure was attributed to disturbances in miRNA and protein expression. Indeed, miRNA-7, miRNA-17, and miRNA-214 alterations have been demonstrated to play a pathogenic role in lung cancer and to be related to radon exposure [88].

**Figure 3:**
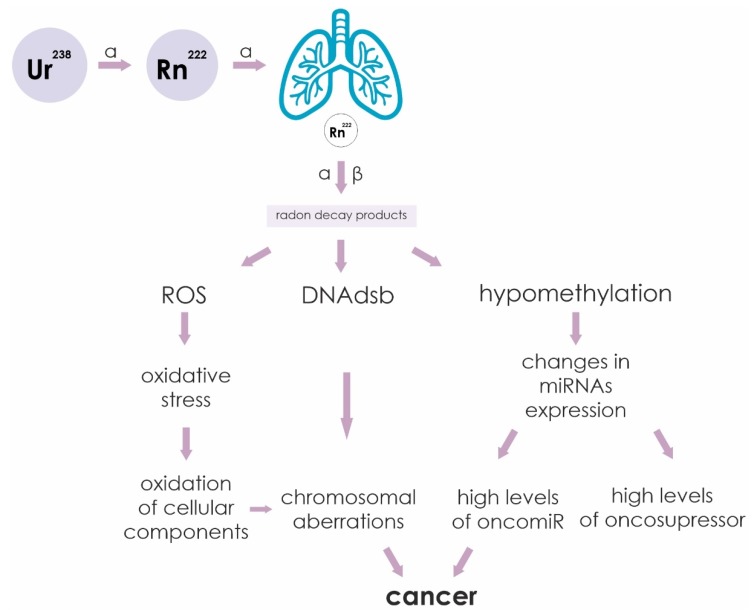
Sequence of events leading from irradiation of cells by alpha and beta particles to cancer. Ionizing radiation causes various cellular damage: double-strand DNA breaks (DNAdsb), formation of reactive oxygen species (ROS) and hypomethylation of CpG sites. Increased ROS leads to oxidation of cellular components as a result of oxidative stress. The formation of double-stranded DNA breaks leads to chromosomal rearrangements. Hypomethylation of miRNA gene promoters leads to a change in their profile. All these changes in the cell are negative factors that can lead to malignant transformation of cells into cancer.

The sequence of events leading from irradiation of cells by alpha particles to cancer is outlined in Figure 3. Ionizing radiation causes various cellular damage: double-strand DNA breaks (DNAdsb), formation of reactive oxygen species (ROS) and hypomethylation of CpG sites. Increased ROS leads to oxidation of cellular components as a result of oxidative stress. The formation of DNAdsb leads to chromosomal rearrangements. Hypomethylation of miRNA gene promoters leads to a change in their profile. All these changes in the cell are negative factors that can lead to malignant transformation of cells into cancer.

Ionizing radiation that affects cells changes the pattern of DNA methylation [89]. It was shown that residential radon exposure influenced DNA methylation profile in an exposure-dependent manner [90].

We are able to assume that exposure to radon (ionizing radiation) affects the profile of miRNA expression by removing methyl groups from oncomiR promoters. The role of ionizing radiation in the regulation of miRNA expression was shown in a study carried out by Shin et al., who studied the miRNA expression profile in lung adenocarcinoma cells (A549) in response to radiation. It was determined that more than twofold changes in the levels of miRNA expression. Overexpression of these miRNAs is aimed at regulating the cellular response caused by the action of ionizing radiation [91]. Additionally, miR-9 overexpression increased the radio-sensitivity of A549 cells by inhibiting cell activity and migration, and by increasing apoptosis. Accordingly, the promoter methylation status of the microRNA-9 gene increased in response to ionizing radiation [92].

It is known that *K-ras*, as oncogene, is closely related to tumor development, cell signaling, and intracellular metabolism, and is identified to correlate with increased ROS production and reduced mitochondrial activity. Emerging data show that inhibition of tumor growth may occur via suppression of *K-ras* expression by miRNA let-7a. let-7 and *K-ras* alteration have been reported to occur in rat lung tissues and human bronchial epithelial cells upon exposure to radon [93].

Chronic exposure to radon induces oxidative damage, let-7 downregulation and consequent mutant *K-ras* over-expression both in human bronchial epithelial cells [93].

The regulation of cell death in radon-induced cell damage showed an increase in the expression of miR-34a exposed to radon for a long period of time. Overexpression of miR-34a stimulated apoptosis due to increased expression of the pro-apoptotic Bax protein and a simultaneous decrease in the expression of anti-apoptotic proteins BCL-2 and PARP-1. BEAS-2B cells were transfected with miR-34a, this led to the activation of apoptosis, a decrease in apoptotic effect was observed with the introduction of the miR-34a inhibitor. The results of this study indicate that miR-34a is a cancer suppressor in radon-induced oncogenesis [10].

Exposure to radon induces dysregulation in miRNA level, as shown in an experiment by Cui et al. in human bronchial epithelium cells (BEAS2B) exposed for up to 20 generations (Rn5-1 and Rn5-20). Molecular mechanisms indicating malignant transformations were evaluated by apoptosis level and change miRNA profiles. Analysis of differential miRNA expression in Rn5-1 cells showed an increase of 163 (hsa-miR-16-5p, hsa-miR-15b-5p hsa-miR-15a-5p, hsa-miR-23b-3p, hsa-miR-19b -3p, and hsa-miR-125b-5p) and decrease of 155 miRNA molecules (let-7b-3p, hsa-miR-194-3p, hsa-miR-373-5p hsa-miR-124-3p, hsa-miR-369-3p, and hsa-miR-652-5p). Over expression of 30 different miRNAs (main miRPlus-E1067, hsa-miR-146b-3p, and hsa-miR-146b-5p) and profile of 28 molecules (hsa-miRPlus-F1147, hsa- miRPlus-F1104, and hsa-miR-375) was reduced in Rn5-20 cells compared to control cells. A high level of expression of mature hsa-miR-146b-5p and hsa-miR-744 was shown in cell lines Rn5-1 and Rn5-20 [16].

**Figure 4:**
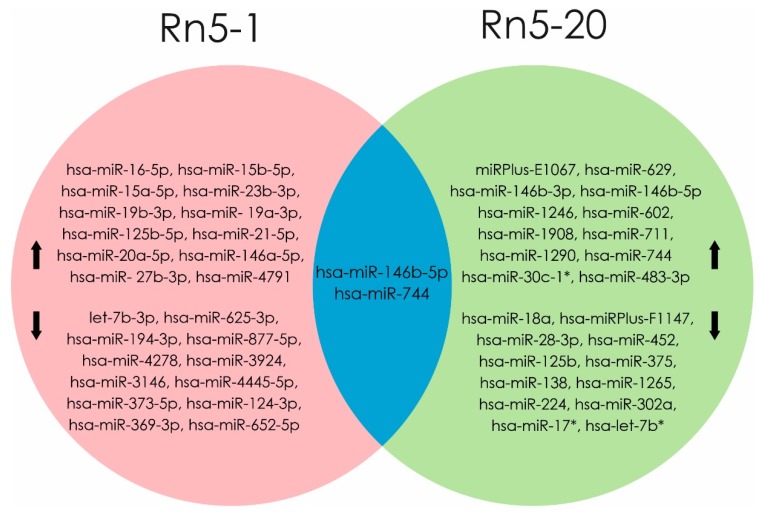
miRNAs profile were changed when exposed to radon in Rn5-1 cells (in the pink area). miRNAs profile were changed when exposed to radon in Rn5-20 cells (in the green area). At the intersection of these regions (blue part) indicated miRNAs, the level of which increased in both cell lines (Rn5-1 and Rn5-20). (Data from [16]). microRNAs that are associated with the development of radon-induced lung cancer are shown in bold text

Multiple and chronic low-level radon exposure induced malignant transformation of BEAS2B cells, in conjunction with differential expression profiles of miRNA and alterations in signal pathways related to regulation of cell proliferation, differentiation, and malignant transformation. Their results suggest that there is miRNAs involvement in progression of tumorigenesis following radon irradiation [16]. The identity of miRNA altered by radon in human bronchial epithelial cells is reported in Figure 4.

Li et al. demonstrated that miR-146b-5p can inhibit the proliferation of lung tumor cells, as indicated by a decrease in the ability to form colonies (H1299). miR-146b-5p suppresses the expression of AUF1, TRAF6, and MMP6 in H1299 cell lines. A high level of miR-146b-5p inversely correlates with a decrease in TRAF6 in NSCLC cells [94].

Indeed, the decreased expression of miR-744-5p in NSCLC cell lines is shown. miR-744-5p overexpression led to inhibition of proliferation, colony formation and invasion of NSCLC cells in vitro. PAX2 was validated as a functional target for miR-744-5p in NSCLC. miR-744-5p inhibits PAX2 protein synthesis, over expression of miR-744-5p stimulates the growth and invasion of cancer cells [95]. Residential radon exposure was associated with DNA methylation in an exposure-dependent manner. Although chance and residual confounding cannot be excluded, the identified associations may show biological mechanisms involved in early biological effects from radon exposure [89].

Exposure to high doses of radon leads to lower levels of miR-194-3p. Over expression of miR-194 inhibits cancer cell metastasis in lung oncology. The targets of miR-194 are BMP1 and p27 kip1. Down-regulated BMP1 suppresses activity of TGFβ, in turn, MMP2 and MMP9 activities are decreased. In addition, miRNA-194-induced suppression of p27 kip1 activates the RhoA signaling pathway, causing enhanced development of actin stress fibers and impaired cancer cell migration [96].

Recently, it was shown that miR-19b-3p is associated with a risk of developing pulmonary neoplasia. The levels of miR-19b-3p in blood plasma of lung cancer patients were significantly higher compared to the healthy control group. In NSCLC cell lines miR-19b potentiates cell proliferation and inhibit apoptosis by targeting PP2A B subunit PPP2R5E and BCL2L11 [97]. However, statistically significant differences in the expression level of miR-19b-3p in cancer patients exposed to prolonged exposure to radon and living in areas with low radon levels were not found [98]. Based on the obtained results, it was concluded that miR-19b-3p is not suitable as the genetic marker of radon exposure.

**Figure 5:**
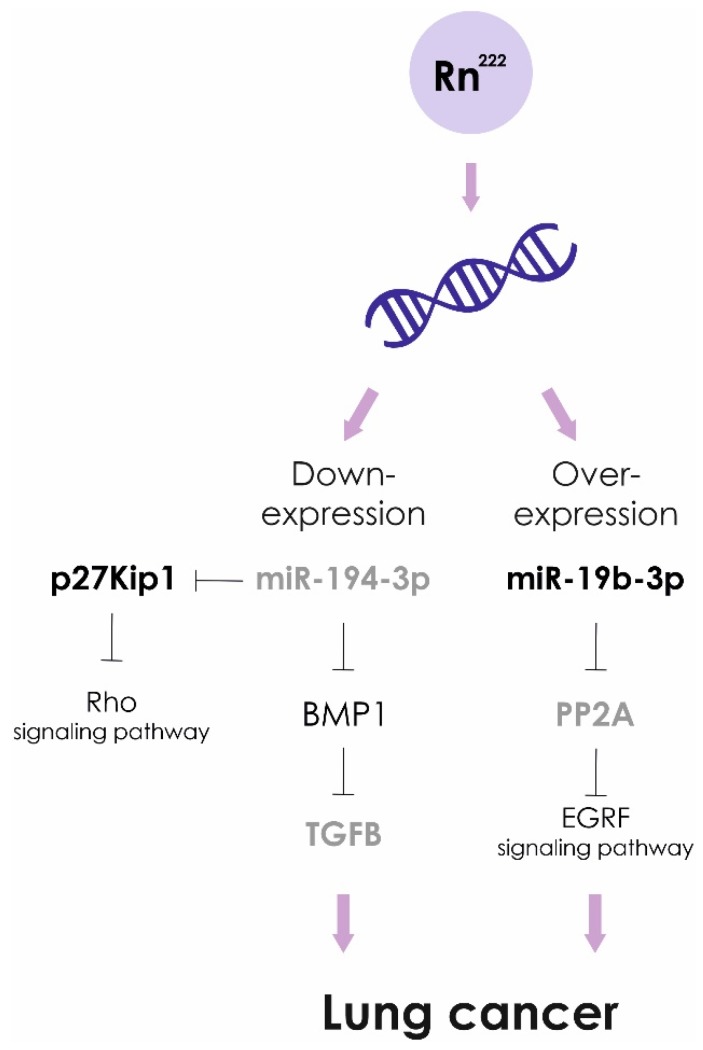
Radon exposure and lung cancer. Molecular pathogenetic mechanisms.

An overview of the main cytogenetic and “omics” biomonitoring studies focused on the effect of radon on various biomarkers including microRNAs is reported in Figure 5 and Table 1.

## 8. Conclusions

Analysis of literature allows us to conclude that alterations of miRNA expression occur upon exposure to radon. These events are initially adaptive, being aimed at inhibiting neoplastic transformation and cancer cell proliferation. However, in the case of long-term exposure to radon, miRNA alterations become leads to the development of cancer. Accordingly, it is difficult to establish a straight miRNA signature reflecting radon exposure. It is more conceivable that miRNA signature can be used retrospectively to evaluate the possible association of a lung cancer with radon exposure. Several etiologic factors have been proposed for the development of lung cancer, including exposure to radon, cooking fumes, asbestos, heavy metals, and environmental tobacco smoke, and inherited genetic susceptibility. miRNA analysis could contribute to highlight the relative significance of radon exposure in the development of lung cancer.

## Figures and Tables

**Table 1:** Main cytogenetic and “omics” biomonitoring studies focused on the effect of radon on various biomarkers including microRNAs.

Study	Study Type	Study Type Methods	Total Size Samples	Location Detail of Exposure	Endpoint	Results
Wu et al., 2019 [10]	In vitro	qRT-PCR, flow cytometry, western blot analysis	Human bronchial epithelial BEAS-2B cell culture	Bronchial epithelial BEAS2B cells underwent exposure to radon for 30 min for 1, 5, 10, 15 and 20 days (Rn1, Rn5, Rn10, Rn15, Rn20).	miR-34a expression, apoptosis	miR-34a upregulation. PARP-1 and Bcl-2 downregulation and Bax upregulation in Rn20 cells.
Cui et al., 2013 [16]	In vitro	RNA isolation, microRNA microarray	Human bronchial BEAS2B cell line were cultured in LHC-8 medium	BEAS-2B cells were exposed to radon for 10, 20, 30 min at a concentration of 20,000 Bq/m^3^ during fifth passage for 1 (Rn5-1) and 20 generations (Rn5-20)	Modification of miRNA profile expression	163 miRNA upregulated and 155 miRNA downregulated in Rn-5-1 cells. 30 miRNA upregulated and 28 miRNA downregulated in Rn-5-20 cells
Meenakashi et al., 2017 [29]	In vivo	Micronucleus assay	25 healthy smokers and 25 healthy non-smokers (males)	Blood samples were exposed to radon gas with doses ranging between 0.3–12.6 mGy	Nucleoplasmic bridges as a biomarker of early DNA damage induced by radon	Radon exposure increased DNA damage in smokers compared to non-smokers
Zölzer et al., 2013 [36]	In vivo	Modified micronucleus-centromere test	84 uranium miners and 52 control persons	Mine workers exposed to 35–90 mSv	Micronuclei in blood lymphocytes	Uranium-exposed subjects had higher micronuclei frequency than non-exposed
Rosenberger et al., 2019 [53]	In vivo	Infinium OncoArray-500K	15,077 cases (lung cancer) and 13,522 controls, including 463 former uranium miners (61 cases:402 controls)	49 of 15 077 (0.3%) LC cases and 259 of 13 522 cancer-free controls (1.9%) had been occupationally exposed by a high cumulative dose exposure to radon and its progeny (WLM > 50)	occupational radon exposure was categorized into ≤50 (“unexposed”) and >50 WLM (“exposed”) as a threshold for significant elevated relative lung cancer risk	Genes belonging to the Gene Ontology term “DNA dealkylation involved in DNA repair” (GO:0006307; *p* = 0.0139) or the gene family HGNC:476 “microRNAs” (*p* = 0.0159) were enriched with LD-blockwise significance
Bulgakova et al., 2019 [57]	In vivo	DNA isolation, PCR-RFLP	44 radon-induced lung cancer patients and 41 lung cancer patients without high level of radon exposure and 42 age/sex-matched healthy controls	The average equivalent equilibrium radon volume activity (EEVA) for radon-induced lung cancer patients was 307.6 Bq/m^3^. The EEVA in the lung cancer patients living on the territory with a low level of radon were 40.6 Bq/m^3^	Polymorphism *TP53* Arg72Pro (rs1042522) was showed a significantly higher risk of radon-induced lung cancer	Arg/Pro and Pro/Pro variants conferred an odds ratio (OR) of 6.95 (95 % confidence interval (CI) 2.41–20.05) and 1.45 (95 %CI 0.46–4.64), respectively. Individuals with Arg/Pro variant of *TP53* gene exposed to high level of radon have a high risk of lung cancer (OR = 8.6; 95% CI 2.6–28.59) compared with people living in areas with a low level of radon
de Vocht et al., 2019 [90]	In vivo	Illumina Infinium HumanMethylation450 BeadChip	14,541 pregnant women with expected delivery dates between April 1991 and December 1992, which resulted in 14,062 live births of which 13,988 children were alive at 1 year of age	Estimates of potential radon exposure were based on long-term radon measurements from 479,000 homes across Great Britain and provided with a spatial resolution of 75-metre buffers as the percentage of dwellings exceeding the 200 Bq/m^3^. Radon Action Level in 6 classes: 1 (0–1%), 2 (1–3%), 3 (>3–5%), 4 (>5–10%), 5 (>10–30%) and 6 (>30–100%).	Once each residential address had a radon potential exposure class assigned, time spent at each address was calculated. This was merged with ARIES sample prevision dates, allowing time-weighted average potential radon exposures to be calculated up to the “mothers at middle age”, “children at 7” and “children at 15/17” sample extraction time points.	Average potential exposure to radon was associated in an exposure-dependent manner with methylation at cg25422346 in mothers during pregnancy, with no associations at middle age. For children, radon potential exposure was associated in an exposure-dependent manner with methylation of cg16451995 at birth, cg01864468 at age 7, and cg04912984, cg16105117, cg23988964, cg04945076, cg08601898, cg16260355 and cg26056703 in adolescence.
Chen et al., 2015 [93]	In vitro	qRT-PCR, western blot	Human bronchial epithelial (HBE) cells	Each time 1 × 106 HBE cells were seeded on transwell membrane and exposed to radon at the concentration of 20,000 Bq/m^3^ for 20 min. The exposure was repeated for 5 times (HR-5) or 10 times (HR-10)	let-7 microRNA and K-ras may be of potential markers in early diagnosis and therapy of radon-induced lung cancer	Down-regulation of let-7 and up-regulation of K-ras were revealed both in mRNA and in protein level in lung tissue of rats and HBE cells exposed to radon

HBE (Human Bronchial epithelial) cells; EEVA (Equivalent equilibrium radon volume activity); OR (Odds Ratio); WLM (Working Level Month); BEAS (Human bronchial epithelial).
